# Farmers’ knowledge of Johne’s disease and opinions of the Irish Johne’s Control Programme: results of an online survey answered mostly by young farmers

**DOI:** 10.1186/s13620-023-00260-x

**Published:** 2023-10-20

**Authors:** Louise Horan, John F. Mee, Niamh L. Field, Siobhán W. Walsh, Ainhoa Valldecabres

**Affiliations:** 1 Animal and Bioscience Research Department, Animal & Grassland Research and Innovation Centre, Teagasc, Moorepark, Fermoy, Co. Cork, P61 P302 Ireland; 2 Department of Land Sciences, South East Technological University, Cork Road Campus, Waterford, Co. Waterford, X91 K0EK Ireland

**Keywords:** Infectious disease, Questionnaire, Voluntary control programme, IJCP

## Abstract

A voluntary control programme for Johne’s disease, the Irish Johne’s Control Programme (IJCP) has been implemented in Ireland since 2017. The objective of this observational study was to assess Irish beef and dairy farmers’ Johne’s disease knowledge, implemented management practices and IJCP opinions. A questionnaire open to dairy and beef farmers was distributed via social media and email. In total 126 responses were used for this study; these responses came from mostly young farmers (18–25 years old) and represent a small proportion of the total number of dairy and beef farmers in Ireland whose average age is 55.

Most respondents claimed to know what Johne’s disease was (73%; 92/126) and associated the disease to loss of body condition (68%; 78/114) and diarrhoea (59%; 67/114). Twenty-eight respondents (mostly dairy farmers; 22/28) reported positive cases in their premises. And 38% reported to implement management practices to prevent Johne’s disease transmission within or into their herd (i.e. management of milk for calf consumption and isolation of Johne’s test-positive or newly purchased stock; 47/124).

Eighteen percent (22/125) of respondents were, at the time of questionnaire or previously, members of the IJCP. The main benefits reported by some of the participating farmers were identification of positive cases (29%; 4/14), and management of milk for calf consumption (21%; 3/14). While the main disadvantage was inaccurate testing methods (50%; 10/20). The main reasons reported for the lack of participation in the IJCP were not being aware of the programme (52%; 53/102) and not having a Johne’s disease problem on the farm (48%; 49/102).

In conclusion, this study suggests that while young farmers are aware of Johne’s disease, their participation in the IJCP is limited and could benefit from further promotion. Studies representing the wider farming community in Ireland are warranted to gather non-biased input and contribute to Johne’s disease control in Ireland.

**Supplementary Information:**

The online version contains supplementary material available at 10.1186/s13620-023-00260-x.

## Main text

Johne’s disease is an infectious, enteric, wasting disease caused by the bacterium *Mycobacterium avium* subspecies *paratuberculosis* (MAP) [1]. Following a period of subclinical infection, the disease is characterised by severe weight loss, diarrhoea and premature culling, and can impact productivity of infected animals, regardless of whether clinical infection is observed or not [2–4]. In addition, although no evidence of a causal link has been established, MAP has been associated with Crohn’s disease in humans [5]. Given these issues, Johne’s disease control is a priority for Irish animal health stakeholders [6, 7].

Hence, the Irish Johne’s Control Programme was established in 2017. It is coordinated by Animal Health Ireland (AHI) and is a voluntary programme aiming to encourage Johne’s disease control in the Republic of Ireland. This programme promotes testing for Johne’s disease, assists farmers in maintaining a negative herd status, aids the reduction of herd infection, helps optimise overall calf health and farm biosecurity and provides reassurance of Johne’s disease control to the food market [8]. Expert opinion and recommendations typically provide the basis for the creation of most animal disease control programmes [9]. However, end-user (i.e. farmer) input is essential so that the programme is easily implemented, convenient and therefore more widely accepted. Hence, the objectives of this study were to gain insight into dairy and beef farmers’ Johne’s disease knowledge, implemented management practices, and to elicit the IJCP opinions of farmers who were either participants or non-participants of the IJCP.

An anonymous questionnaire composed of 14 questions (11 multiple choice and 3 open-ended type questions) divided into three sections was designed (see Additional file 1). The first section gathered general demographic information of the respondent and their herd, the second section assessed farmers’ general knowledge of Johne’s disease, and the third section addressed farmer’s opinion of the IJCP. The 14 questions were uploaded onto Google Forms (Google, Mountain View, CA, USA). The on-line questionnaire was pilot-tested with three dairy farmers and two beef farmers and modified based on their suggestions. The final questionnaire was posted onto the first author’s personal social media platforms (Instagram and Facebook) in February 2022. Additionally, a link to the questionnaire was sent via email to 66 beef and dairy farmers in two farmer discussion groups (one with 49 dairy and beef farmers and one with 17 dairy farmers only, chosen based on proximity to the first author). Farmers were not offered any incentive to complete the survey. The questionnaire was open for five days; one social media reminder post and one reminder email were sent both on day three. Once the questionnaire was closed the results were exported from Google Forms to Excel (Microsoft Corp., Redmond, WA, USA) where the responses were collated and descriptive statistics were generated.

In total, 128 respondents completed the questionnaire. Responses were checked for abnormalities and signs of bot activity before data analysis. First timestamps were assessed to identify abnormally fast responses and second, open-ended answers were assessed for any illogical or repeated statements [10]. One response was excluded from data analysis for not indicating an enterprise type (questionnaire was opened to beef and dairy farmers only); and another response was excluded for offering uninterpretable answers to all open-ended questions. Therefore a denominator of 126 was used where all respondents answered a question; denominators < 126 indicate missing answers to the specific question. Percentage of respondents per answer followed by number of respondents per answer over the total number of respondents per question are provided for each answer in the result sections below and are presented in Additional file 1. The 95% confidence intervals associated with the proportions were calculated using an online calculator and are included in Additional file 1 [11].

### Section one: participant and herd information

Half (50%; 63/125) of respondents were < 26 years old and the majority of respondents were dairy farmers [57% (72/126); beef: 43% (54/126)]. Respondents had > 200 cows (29%; 36/126), 50 to 100 cows (28%; 35/126), 100 to 200 cows (26%; 33/126), or < 50 cows (18%; 22/126).

### Section two: farmers’ general knowledge of Johne’s disease

**Fig. 1:**
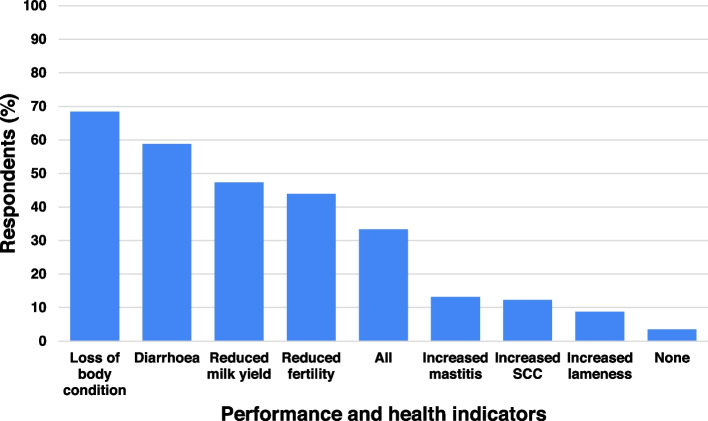
Reported health and performance indicators believed to be associated with Johne’s disease by 114 questionnaire respondents. 'All' represents all of the enumerated performance and health indicators while 'None' represents none of these indicators

The majority (73%; 92/126) of respondents reported knowing what Johne’s disease was, 19% (24/126) had ‘some idea’ and 8% (10/126) had no idea. A total of 114 out of the 116 respondents who knew, or had ‘some idea’ what Johne’s disease was, provided answers for the follow up question regarding performance and health indicators they associate with the disease. The majority of respondents listed loss of body condition (68%; 78/114) and diarrhoea (59%; 67/114) as clinical signs they associate with Johne’s disease; a minority listed other clinical signs which are presented in Fig. 1 and listed in Additional file 1.

In total, 28 respondents (23%; 28/124) reported that cattle in their herd had tested positive for Johne’s disease by any test method. More dairy (22/28) than beef (6/28) farmers reported this. Forty-one respondents provided answers to the question regarding clinical signs and production impacts noticed in Johne’s disease-positive animals. Of those, 42% (17/41) reported having witnessed no clinical signs in the positive cows.

**Fig. 2:**
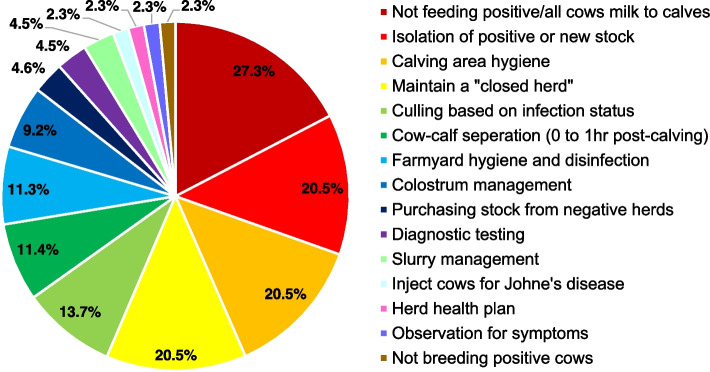
Management practices reported to be implemented by 44 respondents with the goal of preventing Johne's disease transmission. Management practices have been categorised for data summarisation and are listed in order of increasing reported frequency

Thirty-eight percent (47/124) of respondents reported that they had implemented management practices to prevent the transmission of Johne’s disease within or into their herd. The follow-up open-ended question which addressed these management practices was answered by 44 out of these 47 respondents. Maintaining good calving area hygiene (21%; 9/44) and maintaining a closed herd (21%; 9/44) were the two most commonly reported practices. Upon categorising the answers from this open-ended question, management of milk for calf consumption was the most common management practice (Fig. 2). Individual reported management practices are presented in Fig. 2 and listed in Additional file 1.

### Section three: knowledge and opinions about the IJCP

The majority (82%; 103/125) of respondents were not members of the IJCP, and over half of these respondents had not heard of the IJCP (52%; 53/102; one non-member did not answer this question). Twenty-two respondents were either members at the time of the questionnaire or had previously been members of the programme (21 of which were dairy farmers), hence results from questions regarding benefits and disadvantages of the IJCP could only be based on a maximum of 22 responses. Among the respondents that participated in the IJCP, 32% (7/22) reported to have experienced a positive Johne’s disease case in their premises, and 73% (16/22) implemented preventative measures to reduce Johne’s disease transmission.

The respondents who were members of the IJCP listed the following as benefits of the membership: identification of sub-clinical cases of Johne’s disease (21%; 4/19), knowing cows’ infection status before feeding their milk to calves (16%; 3/19), “peace of mind” (11%; 2/19), facilitated culling decisions (11%; 2/19) and improved herd health (11%; 2/19). However, five members reported that they could identify no benefits to the programme. The respondents who were members of the IJCP listed the following as disadvantages of membership: inaccuracy of testing methods (50%; 10/20) and labour and management-related issues (25%; 5/20). The full list of reported advantages and disadvantages is provided in Additional file 1.

The main reasons listed for not joining the IJCP were: not being aware of the programme (52%; 53/102), not having Johne’s disease in the herd (48%; 49/102), and not seeing the advantages of the programme (7%; 7/102). The full list of reported reasons is presented in Additional file 1.

To the best of our knowledge, this study is the first to assess Irish beef and dairy farmers’ who participated or not in the IJCP general Johne’s disease knowledge and opinions of the programme. Similar approaches have been used globally to assess other animal health control programmes [12–14]. This study, allowed farmers to freely express their opinions without the restriction of a “multiple choice only” format or the influence of the physical presence of an interviewer. The study provides a valuable source of information to gain insight into Irish cattle farmers Johne’s disease knowledge and attitudes towards the IJCP. However, study limitations have to be considered when interpreting our results. First, given the distribution methods used, the questionnaire reached farmers that are not fully representative of the Irish cattle farmers (i.e. young farmers, social media users, email users and discussion group members), thus it has to be acknowledged that the presented results are based on a bias sample. For instance, over half of the respondents to this survey were between 18 and 25 years old. This is a much younger population than the national average Irish dairy farmer which is 55 years old [15]. Second, with the questionnaire being anonymous we could not corroborate the provided answers with national databases. And third, we only reached to a small sample of Irish beef and dairy farmers (Dairy: 15,319 farms; Beef: 48,227 farms [15]), as well as a small proportion of IJCP participants (2,206 members in September 2023; personal AHI communication), consequently limiting the power of the study. Further studies designed to reach the wider Irish farming community (i.e. using mobile text messaging or mail distribution methods) are warranted to generate results which could be extrapolated to the majority of dairy and beef farmers in Ireland.

The major part of respondents were aware of what Johne’s disease was and it was apparent that respondents associated clinical signs (i.e. loss of body condition and diarrhoea), more than performance impacts, with the disease. A higher percentage of dairy farmers had identified positive cases within their herd than that of beef farmers. However, beef farms have only been accepted as participants in the IJCP since 2020; and as a result testing on beef farms was most likely less common than on dairy farms who have been accepted into the programme since 2017. A significant percentage of respondents reported to observe no clinical signs of Johne’s disease in test-positive cattle. This may have been reflected in farmer’s opinions of the IJCP; respondents that did not observe any negative performance or health indicators may have reported seeing no benefit to the programme as they could not observe a physical change in their herd performance or health.

The final results of this study were shared with AHI and may be considered for the IJCP development. Based on this study, the use of social media could help promote the IJCP among younger farmers. In order to decrease Johne’s disease prevalence nationally, it is essential that a large percentage of farmers participate in the IJCP. Discussion groups and other farmer educating activities should emphasise appropriate control measures to prevent transmission of the disease and the impact of Johne’s disease on cattle productivity as farmers were less engaged and aware of these areas.

### Supplementary Information

**Additional file 1. **Questionnaire description and results.

## Data Availability

All data generated or analysed during this study are included in this published article (Additional file 1).

## Abbreviations

AHI: Animal Health Ireland
IJCP: Irish Johne’s Control Programme
MAP: *Mycobacterium avium* Subspecies *paratuberculosis*
SCC: Somatic cell count
