# Supplementary material for: Farmers’ knowledge of Johne’s disease and opinions of the Irish Johne’s Control Programme: results of an online survey answered mostly by young farmers
Source: Ir Vet J. 2023 Oct 20;76:31. doi: 10.1186/s13620-023-00260-x (PMC10588086; doi:10.1186/s13620-023-00260-x)
Supplement: Supplementary file 1 — Additional file 1. Questionnaire description and results. [file 13620_2023_260_MOESM1_ESM.docx]

**Additional file 1. Questionnaire description and results**

|  |  | **Outcome** | |
| --- | --- | --- | --- |
| **Question** | **Response** | **Fraction^1^** | **Proportion (95% confidence interval)** |
| What age group are you in? | 18-25 | 63/125 | 50.4% (41% - 59%) |
|  | 26-35 | 35/125 | 28.0% (20% - 37%) |
|  | 36-55 | 24/125 | 19.2% (13% - 27%) |
|  | 56-75 | 3/125 | 2.4% (1% - 7%) |
|  | 75+ | 0/125 | 0.0% (0% - 3%) |
|  | | |  |
| What enterprise do you operate on your farm? | Dairy | 72/126 | 57.1% (48% - 66%) |
|  | Beef | 54/126 | 42.9% (34% - 52%) |
|  | | |  |
| How many beef/dairy stock do you own? | 0-50 | 22/126 | 17.5% (11% - 25%) |
|  | 50-100 | 35/126 | 27.8% (20% - 36%) |
|  | 100-200 | 33/126 | 26.2% (19% - 35%) |
|  | 200-300 | 21/126 | 16.7% (11% - 24%) |
|  | 300+ | 15/126 | 11.9% (7% - 19%) |
|  | | |  |
| Are you aware of what Johne’s disease is? | Yes | 92/126 | 73.0% (64% - 81%) |
|  | Some idea | 24/126 | 19.0% (13% - 27%) |
|  | No | 10/126 | 7.9% (4% - 14%) |
|  | | |  |
| If yes, then what health and production impacts would you associate with the disease? | Loss of body condition | 78/114 | 68.4% (59% - 77%) |
|  | Diarrhoea | 67/114 | 58.8% (49% - 68%) |
|  | Reduced milk yield | 54/114 | 47.4% (38% - 57%) |
|  | Reduced fertility | 50/114 | 43.9% (35% - 53%) |
|  | All of the above | 38/114 | 33.3% (25% - 43%) |
|  | Increased mastitis | 15/114 | 13.2% (8% - 21%) |
|  | Increased somatic cell count | 14/114 | 12.3% (7% - 20%) |
|  | Increased lameness | 10/114 | 8.8% (4% - 16%) |
|  | None of the above | 4/114 | 3.5% (1% - 9%) |
|  | | |  |
| Have you had cattle test positive for Johne’s disease on your farm? | No | 96/124 | 77.4% (69% - 84%) |
|  | Yes | 28/124 | 22.6% (16% - 31%) |
|  | | |  |
| If yes, what symptoms and production impacts did you notice in the Johne’s positive animals (if any)? | Loss of body condition | 21/41 | 51.2% (35% - 67%) |
|  | Diarrhoea | 18/41 | 43.9% (28% - 60%) |
|  | None of the above | 17/41 | 41.5% (26% - 58%) |
|  | Reduced milk yield | 9/41 | 22.0% (11% - 38%) |
|  | Reduced fertility | 6/41 | 14.6% (6% - 29%) |
|  | Increased mastitis | 5/41 | 12.2% (4% - 26%) |
|  | Increased somatic cell count | 5/41 | 12.2% (4% - 26%) |
|  | All of the above | 5/41 | 12.2% (4% - 26%) |
|  | Increased lameness | 4/41 | 9.8% (3% - 23%) |
|  | | |  |
| Do you actively take any precautions on your farm to prevent the spread of Johne’s disease specifically? | No | 77/124 | 61.9% (53% – 71%) |
|  | Yes | 47/124 | 37.9% (29% – 47%) |
|  | | |  |
| If yes, please outline these precautions. | Maintaining a clean calving area | 9/44 | 20.5% (10% – 35%) |
|  | Maintaining a closed herd | 9/44 | 20.5% (10% – 35%) |
|  | Culling positive cows | 5/44 | 11.4% (4% – 25%) |
|  | Discarding positive cows milk | 4/44 | 9.1% (3% – 22%) |
|  | Calves only receive milk from negative cows | 4/44 | 9.1% (3% – 22%) |
|  | Separate calving areas for positive and negative cows | 4/44 | 9.1% (3% – 22%) |
|  | Replacement heifer calves get milk replacer only | 3/44 | 6.8% (1% – 19%) |
|  | Water troughs are cleaned | 2/44 | 4.5% (1% – 15%) |
|  | Diagnostic testing | 2/44 | 4.5% (1% – 15%) |
|  | Calf is separated from cow immediately after calving | 2/44 | 4.5% (1% – 15%) |
|  | Slurry management (i.e. not letting cattle graze field after recent slurry application, not using slurry spreading equipment from other farms) | 2/44 | 4.5% (1% – 15%) |
|  | Footbaths | 2/44 | 4.5% (1% – 15%) |
|  | Isolation area for sick or newly purchase animals | 2/44 | 4.5% (1% – 15%) |
|  | Purchasing colostrum only from herds with negative infection status | 1/44 | 2.3% (0% – 12%) |
|  | Only purchasing animals with a known infection status | 1/44 | 2.3% (0% – 12%) |
|  | Housing positive cows separate to negative cows | 1/44 | 2.3% (0% – 12%) |
|  | Positive cows are isolated from negative cows for a few days after calving | 1/44 | 2.3% (0% – 12%) |
|  | Each calf receives its own dams colostrum | 1/44 | 2.3% (0% – 12%) |
|  | Using a herd health plan | 1/44 | 2.3% (0% – 12%) |
|  | Replacement heifers kept in a separate shed to the milking herd | 1/44 | 2.3% (0% – 12%) |
|  | Not breeding positive cows | 1/44 | 2.3% (0% – 12%) |
|  | Separating cow from calf within half an hour after calving | 1/44 | 2.3% (0% – 12%) |
|  | Colostrum management | 1/44 | 2.3% (0% – 12%) |
|  | Separating cow from calf as soon as possible | 1/44 | 2.3% (0% – 12%) |
|  | Different boots for wearing specifically in the calf shed | 1/44 | 2.3% (0% – 12%) |
|  | Observing the herd for Johne’s disease symptoms | 1/44 | 2.3% (0% – 12%) |
|  | Not importing colostrum from other farms | 1/44 | 2.3% (0% – 12%) |
|  | Separating cow from calf within an hour after calving | 1/44 | 2.3% (0% – 12%) |
|  | Cull cows with suspected infection | 1/44 | 2.3% (0% – 12%) |
|  | Feed milk replacer to all calves only | 1/44 | 2.3% (0% – 12%) |
|  | Inject cows for Johne’s disease prevention | 1/44 | 2.3% (0% – 12%) |
|  | Only purchasing animals from “reliable farms” | 1/44 | 2.3% (0% – 12%) |
|  | | |  |
| Have you heard of the Irish Johne’s control programme? | No | 68/126 | 54.0% (45% – 63%) |
|  | Yes | 58/126 | 46.0% (37% – 55%) |
|  | | |  |
| Have you ever participated in the Irish Johne’s control programme? | No, I have never been part of this programme | 103/125 | 82.4% (75% – 89%) |
|  | Yes, currently in the programme | 16/125 | 12.8% (8% – 20%) |
|  | Yes, previously in the programme | 6/125 | 4.8% (2% – 10%) |
|  | | |  |
| If yes, what benefits have you seen from being in the programme? | None | 5/19 | 26.3% (9% – 51%) |
|  | Identifying sub-clinical cases | 4/19 | 21.1% (6% – 46%) |
|  | Knowing herd infection status | 3/19 | 15.8% (3% – 40%) |
|  | Not feeding Johne’s disease positive cow’s milk to calves | 3/19 | 15.8% (3% – 40%) |
|  | “Peace of mind” | 2/19 | 10.5% (1% – 33%) |
|  | Knowing which cows to cull before breeding | 2/19 | 10.5% (1% – 33%) |
|  | Improved herd health | 2/19 | 10.5% (1% – 33%) |
|  | Maintaining a negative infection status | 1/19 | 5.3% (0% – 26%) |
|  | Nearing eradication of Johne’s disease | 1/19 | 5.3% (0% – 26%) |
|  | Improved calf management | 1/19 | 5.3% (0% – 26%) |
|  | Selling animals knowing they have tested negative for Johne’s disease | 1/19 | 5.3% (0% – 26%) |
|  | | |  |
| If yes, what disadvantages or areas for improvement have you seen from being in the programme? | Inaccuracy of diagnostic tests | 10/20 | 50.0% (27% – 73%) |
|  | Increased labour required for programme participation | 1/20 | 5.0% (0% – 25%) |
|  | Having to manage the location of slurry spreading on the farm | 1/20 | 5.0% (0% – 25%) |
|  | Having to test all dairy livestock, regardless of whether they are producing milk for human consumption | 1/20 | 5.0% (0% – 25%) |
|  | This programme will not effectively eradicate the disease | 1/20 | 5.0% (0% – 25%) |
|  | Financial cost of diagnostic tests | 1/20 | 5.0% (0% – 25%) |
|  | Ensuring diagnostic testing is completed at least 90 days after bTB testing | 1/20 | 5.0% (0% – 25%) |
|  | This programme is not compulsory | 1/20 | 5.0% (0% – 25%) |
|  | The duration of diagnostic testing on the day | 1/20 | 5.0% (0% – 25%) |
|  | Lack of information about the disease upon joining the programme | 1/20 | 5.0% (0% – 25%) |
|  | None | 1/20 | 5.0% (0% – 25%) |
|  | | |  |
| If you have never been involved in the Irish Johne’s control programme, why not? | I was never aware of the programme | 53/102 | 52.0% (42% – 62%) |
|  | I don’t have a Johne’s disease problem on my farm, therefore don’t see the need to be involved | 49/102 | 48.0% (38% – 58%) |
|  | I do not see the advantages to being in the programme | 7/102 | 6.9% (3% – 14%) |
|  | Farming in the UK | 2/102 | 2.0% (0% – 7%) |
|  | Does not think there are positive cases within the herd | 1/102 | 1.0% (0% – 5%) |
|  | Only recently discovered positive cases within the herd | 1/102 | 1.0% (0% – 5%) |

^1^Fraction: responses/total question respondents
